# Optimized Longitudinal Monitoring of Stem Cell Grafts in Mouse Brain Using a Novel Bioluminescent/Near Infrared Fluorescent Fusion Reporter

**DOI:** 10.1177/0963689717739718

**Published:** 2018-02-02

**Authors:** Laura Mezzanotte, Juvita Delancy Iljas, Ivo Que, Alan Chan, Eric Kaijzel, Rob Hoeben, Clemens Löwik

**Affiliations:** 1 Department of Radiology, Optical Molecular Imaging, Erasmus Medical Center, Rotterdam, the Netherlands; 2 Percuros BV, Enschede, the Netherlands; 3 Max Planck Institute for Metabolism Research, Cologne, Germany; 4 Department of Radiology, Leiden University Medical Center, Leiden, the Netherlands; 5 Department of Molecular Cell Biology, Leiden University Medical Center, Leiden, the Netherlands

**Keywords:** human mesenchymal stem cells, longitudinal study, bioluminescence imaging, fluorescence imaging, CycLuc1 substrate

## Abstract

Biodistribution and fate of transplanted stem cells via longitudinal monitoring has been successfully achieved in the last decade using optical imaging. However, sensitive longitudinal imaging of transplanted stem cells in deep tissue like the brain remains challenging not only due to low light penetration but because of other factors such as low or inferior expression levels of optical reporters in stem cells and stem cell death after transplantation. Here we describe an optimized imaging protocol for sensitive long-term monitoring of bone marrow-derived human mesenchymal stem cells (hMSCs) expressing a novel bioluminescent/near infrared fluorescent (NIRF) fusion reporter transplanted in mouse brain cortex. Lentivirus expressing the luc2-iRFP720 reporter, a fusion between luc2 codon-optimized firefly luciferase (luc2) and the gene encoding NIRF protein iRFP720, was generated to transduce hMSCs. These cells were analyzed for their fluorescent and bioluminescent emission and checked for their differentiation potential. In vivo experiments were performed by transplanting decreasing amounts of luc2-iRFP720 expressing hMSCs in mouse brain, followed by fluorescence and bioluminescence imaging (BLI) starting 1 wk after cell injection when the blood–brain barrier was restored. Bioluminescent images were acquired when signals peaked and used to compare different luc2 substrate performances, that is, D-luciferin (D-Luc; 25 μM/kg or 943 μM/kg) or CycLuc1 (25 μM/kg). Results showed that luc2-iRFP720 expressing hMSCs maintained a good in vitro differentiation potential toward adipocytes, chondrocytes, and osteocytes, suggesting that lentiviral transduction did not affect cell behavior. Moreover, in vivo experiments allowed us to image as low as 1 × 10^5^ cells using both fluorescence and BLI. The highest bioluminescent signals (∼1 × 10^7^ photons per second) were achieved 15 min after the injection of D-Luc (943 μM/kg). This allowed us to monitor as low as 1 × 10^5^ hMSCs for the subsequent 7 wk without a significant drop in bioluminescent signals, suggesting the sustained viability of hMSCs transplanted into the cortex.

## Introduction

Stem cell transplantation in the brain holds promise as a treatment for different neurological disorders such as stroke, brain injury, Alzheimer’s disease, and other neurodegenerative diseases. In fact, stem cell transplantation has the potential to restore function when transplanted cells differentiate and reconstitute neural circuits or when they secrete factors that support the host cells.^1,2^ In addition, human mesenchymal stem cells (hMSCs) have created effects by modulating the immune system^3^ or as carriers for anticancer therapeutics because of their tropism for brain tumors.^4,5^ In particular, autologous bone marrow-derived hMSCs are currently investigated in clinical trials for treatment of brain injury, cerebral palsy in children, and chronic stroke.^6–8^

In the preclinical assessment of cell-based therapies, longitudinal visualization of transplanted stem cells is very important as well as the evaluation of their viability and fate. Commonly, direct labeling of cells before transplantation is employed, using superparamagnetic and/or fluorescent iron oxide nanoparticles (e.g., SPIO) for subsequent magnetic resonance imagining or optical imaging.^9^ Alternatively, genetic modification of hMSCs using reporter genes for optical imaging can be applied.^10^ Optical imaging using gene reporters has the advantage of being straightforward and highly reproducible, and it is associated with relatively low costs compared to nanoparticle tracking. In addition, since gene reporters are stably expressed in stem cells, they prove to be adequate for longitudinal monitoring.^11^

However, sensitive longitudinal imaging of transplanted stem cells in the brain remains challenging not only because of low light penetration in deep tissue but because of other factors such as low or inferior expression levels of optical reporters in stem cells and stem cell death after transplantation.^12^ To date, stem cells can be successfully imaged using bioluminescence imaging (BLI) for at least 2 wk following transplantation in the central nervous system.^13,14^ We have recently shown firefly luciferase (luc2) as a favorable reporter for BLI in the brain thanks to its broad emission spectrum and good kinetics when using D-luciferin (D-Luc) as a substrate.^15^ Also, enhanced sensitivity of brain imaging has been reported with synthetic luciferin CycLuc1 as a substrate.^16^

Besides using BLI, sensitive fluorescence imaging (FLI) with near infrared fluorescent proteins (NIRFPs) has shown promise for in vivo (brain) imaging.^17–19^ Here we describe an optimized imaging protocol for the sensitive long-term monitoring of transplanted hMSCs in mouse brain cortex. Different fusion proteins of luc2 and NIRFPs were designed, and the effect of the reporter expression on stem cell viability and differentiation potentials was evaluated. In addition, the sensitivity of stem cell imaging in the mouse brain was assessed in a longitudinal study.

In particular, 3 pending questions were tackled: (1) Are there differences in imaging sensitivity between BLI and near infrared FLI in brain imaging? (2) Which imaging protocol (D-Luc vs. CycLuc1) ensures the highest sensitivity in the brain? and (3) Is long-term optical monitoring of transduced hMSCs after implantation feasible?

## Materials and Methods

### Cloning

Plasmids pN1-iRFP720 and pN1-iRFP670 were obtained from Addgene (Cambridge, MA, USA). The coding sequences of iRFP720 and iRFP670 were isolated using restriction enzymes AgeI and NotI and cloned into pTurboLuc plasmid replacing TurboFP635.^20^ In the constructs, a linker of 14 amino acids (QSTVPRARDPPVAT) connected the C-terminal region of the luc2 gene to the N-terminal region of the iRFP720 or iRFP670 protein resulting in pluc2-iRFP720 and pluc2-iRFP670, respectively. Next, the luc2-iRFP720 sequence was cut blunt and cloned into the multiple cloning site of lentiviral vector pRLPGK^21^ under the control of the phosphoglycerate kinase (PGK) promoter.

### Cell Culture

Human embryonic kidney (HEK)-293 cells were expanded in Dulbecco’s modified eagle medium (DMEM; Sigma-Aldrich, St. Louis, MO, USA) with 10% fetal bovine serum (FBS; Sigma-Aldrich). The human bone marrow was collected from anonymous surgical waste material in orthopedic surgery. In agreement with the pertinent Leiden University Medical Center (LUMC) guidelines, and in accordance with the Best Practices code of the Dutch Federation of Biomedical Scientific Societies, and based on article 467 of the “Wet op de Geneeskundige Behandelingsovereenkomst,” no informed consent is required for the use of anonymous and nontraceable biological materials, and the institutional ethics committee of the LUMC waived the need for donor consent. We confirm that based on the legislation, the institutional ethics committee of the LUMC waived the need for approval for the use of anonymous surgical waste material. Human mesenchymal stem cells (hMSCs) were derived from the bone marrow (passage 2) and expanded in DMEM containing 10% FBS and 1% penicillin/streptomycin (Thermo Fisher Scientific, Waltham, MA, USA). Basic fibroblast growth factor (bFGF) (0.5 ng/mL; Sigma-Aldrich) was added to the hMSC expansion medium. hMSCs were seeded (3.75 × 10^5^ cells per 75 cm^2^ flask) and incubated at 37 °C in 5% CO_2_ and 95% O_2_. Cells were washed with phosphate-buffered saline (PBS) when 80% confluence was reached and detached with 0.05% trypsin-ethylenediaminetetraacetic acid (EDTA) (Thermo Fisher Scientific) for 5 min at 37 °C.

### Western Blot Analysis

HEK-293 cells (2 × 10^6^), transfected with pluc2-iRFP720, pluc2-iRFP670, and pTurboLuc^20^ plasmids, were lysed using radioimmunoprecipitation assay buffer (Thermo Fisher Scientific), and the total protein concentration was determined utilizing a Pierce bicinchoninic acid (BCA) protein assay kit (Thermo Fisher Scientific). Sodium dodecyl sulfate polyacrylamide gel electrophoresis (SDS-PAGE) was performed with 20 μg of cell extract using a 10% SDS concentration, and proteins were transferred onto a nitrocellulose membrane (GE Healthcare, Chicago, IL, USA). Immunoblotting was performed after membrane blocking using rabbit polyclonal luc2 antibody (1:200; Fitzgerald Industries International, Acton, MA, USA). After overnight incubation, the membrane was washed and incubated for 1 h with a secondary mouse anti-rabbit horseradish peroxidase antibody (1:1,000; Fitzgerald Industries International). Pierce enhanced chemiluminescence substrate (Thermo Fisher Scientific) was utilized for signal detection of the membrane in the Bio-Rad gel doc apparatus (Bio-Rad Laboratories, Hercules, CA, USA).

### Immunofluorescence for Detection of Fusion Proteins in HEK-293 Cells

Transfected HEK-293 cells were cultured on 8-well glass chamber slides (Thermo Fisher Scientific) and transfected with pluc2-iRFP720, pluc2-iRFP670, and pTurboLuc using Fugene HD (Promega, Madison, MA, USA). After 24 h, HEK-293 cells were fixed using 4% formaldehyde. Rabbit polyclonal luc2 antibody (1:100) and anti-rabbit immunoglobulin G (IgG) Alexa Fluor 488 secondary antibody (1:200; Thermo Fisher Scientific) were used for immunofluorescent staining.

### Lentiviral Production and Cell Transduction

Third-generation self-inactivating lentiviral particles were produced using standard protocols as earlier described.^21^ Lentivirus was titrated using a p24 enzyme-linked immunosorbent assay (Cell Biolabs, San Diego, CA, USA). hMSCs were cultured in 6-well plates (7.5 × 10^4^ cells/well) and transduced with a multiplicity of infection of 10 and the use of polybrene (8 μg/mL; Sigma-Aldrich). The next day medium containing the virus was removed, and cells were passaged twice before selection using fluorescent-activated cell sorting (FACS). The 633 nm laser was employed for detection of the iRFP720 protein.

### Proliferation of hMSCs

Non transduced hMSCs at passage 3 were seeded at a density of 8 × 10^3^ cells/cm^2^. The day after, cells were transduced with lentivirus using the protocol described above. Cell viability was assessed after 7 d, by determining the viable/dead ratio with the trypan blue (0.4%) exclusion assay (Thermo Fisher Scientific). The number of viable, labeled cells was calculated in relation to the number of viable, unlabeled cells (processed the same way). The number of viable cells was counted for 3 passages after transduction. In addition, white-field microscopic images were taken 6 d after transduction.

### Differentiation and Analysis of hMSCs

Differentiation medium was made with the supplements and protocol provided by the Mesenchymal Stem Cell Identification Kit (R&D Systems, Minneapolis, MN, USA). For all differentiation experiments, expansion medium was added the day after seeding in the wells and the 15 mL tubes were replaced with differentiation medium to induce differentiation. Medium was changed every 2 to 3 d. After 10 to 14 d, differentiation was terminated, and cells were fixed with 4% formaldehyde. Primary antibodies were included in the Mesenchymal Stem cell Identification Kit (R&D Systems).

#### Adipogenic differentiation

Non transduced hMSCs and transduced hMSCs (hMSC luc2-iRFP720) were seeded in a 6-well plates or 24-well plates with a density of 3.2 × 10^4^ cells/cm^2^. Rosiglitazone (Sigma-Aldrich) with a final concentration of 10 µM was added to the adipogenic differentiation medium. Non transduced hMSCs and luc2-iRFP720 expressing hMSCs were permeabilized and blocked using a solution of 0.3% Triton X-100 (Sigma-Aldrich), bovine serum albumin (BSA) (Sigma-Aldrich), and 1% and 10% normal horse serum (Thermo Fisher Scientific) in PBS. Subsequently, cells were immunostained with anti-monoclonal fatty acid–binding protein (anti-4FABP4; 10 µg/mL), dissolved in the same solution (overnight at 4 °C). After washing, donkey polyclonal secondary goat IgG Alexa Fluor 647 antibody (1:200; Thermo Fisher Scientific) was incubated in the dark for 1 h at room temperature. Staining of nuclei was performed using 4′,6-diamidino-2-phenylindole (DAPI; Sigma-Aldrich). Cells were visualized with the Leica TCS SP8 confocal microscope (Leica Microsystems, Wetzlar, Germany). For histological stainings, hMSC luc2-iRFP720 was cultured in 6-well plates. Adipogenic-differentiated cells were incubated for 10 min at room temperature with filtered 0.3% Oil Red O solution (Sigma-Aldrich).

#### Osteogenic differentiation

To assess the differentiation potential of both non transduced hMSCs and transduced hMSCs (hMSC luc2-iRFP720), cells were seeded in a 6-well plate with a density of 1.6 × 10^4^ cells/cm^2^. Non transduced hMSCs and luc2-iRFP720 expressing hMSCs were immunostained with a mouse antihuman osteocalcin primary antibody. Fixed osteogenically differentiated hMSCs were incubated in the dark for 1 h at room temperature with a donkey anti-mouse IgG Alexa Fluor 488 secondary antibody (1:200; Thermo Fisher). Osteogenic-differentiated cells were stained with filtered 2% Alizarin Red S solution (pH = 4.1 to 4.3; Sigma-Aldrich). Cells were visualized with the Leica TCS SP8 confocal microscope (Leica Microsystems).

#### Chondrogenic differentiation

Both non transduced hMSCs and transduced hMSCs (hMSC luc2-iRFP720) were seeded in a 24-well plate with a density of 5.6 × 10^4^ cells/cm^2^. Alternatively, 2.5 × 10^5^ cells in 1 mL expansion medium were cultured in 15 mL tubes and centrifuged at 200 *g* for 5 min to achieve pellet formation. Non transduced hMSCs and luc2-iRFP720 expressing hMSCs were immunostained with a goat antihuman aggrecan primary antibody following the protocol described above. Chondrogenic differentiated cells cultured in pellets were incubated for 10 to 20 min at room temperature with filtered 0.4% toluidine blue (Sigma-Aldrich) dissolved in sodium acetate buffer (sodium acetate and acetic acid from Sigma-Aldrich; pH = 3.7). For every staining, a negative control of nondifferentiated hMSCs was included. A light microscope with camera was utilized to observe the staining (Leica DM3000, Leica Microsystems).

### Alkaline Phosphatase (ALP) Measurements

Medium samples were taken 14 d after differentiation. ALP activity was measured by adding 120 nM p-nitrophenylphosphate (Thermo Fisher Scientific) in 100 mM glycine/1 mM MgCl_2_/0.1 mM ZnCl_2_ buffer (pH = 10.5; Sigma-Aldrich) and measured for 10 min using a VERSAmax Tunable Microplate Reader at 405 nm (Molecular Devices, Sunnyvale, CA, USA). ALP activity was determined as the slope of the kinetic measurement (mOD [optical density]/min) and corrected for number of cells.

### Relative Oil Red O Accumulation by Spectrophotometry

After fixation, cells were rinsed once with PBS, stained with the Oil Red O working solution for 15 min at room temperature, and then washed 3 times in water. The dye was eluted by adding isopropanol. Cells were placed in a plate shaker for 15 min. One hundred microliter medium per well was removed and transferred to a clean 96-well plate for reading the absorbance (OD) using a VERSAmax Tunable Microplate Reader at 540 nm. The average absorbances of the blank wells and the control and test wells were calculated.

### In Vitro Imaging of hMSCs

A serial dilution of luc2-iRFP720 expressing hMSCs ranging from 1 × 10^5^ cells to 3 × 10^3^ was seeded in triplicate into a 96-well black plate with clear bottom and imaged first using an Odyssey scanner (LICOR Biosciences, Lincoln, NE, USA) at 700 nM to detect fluorescence signals. Then D-Luc (Promega, Madison, WI, USA) at a final concentration of 1 mM was added to the wells and imaged 5 min later using an IVIS Spectrum (Perkin Elmer, Waltham, MA, USA). The following settings were used: open filter, field of view (FOV) C, medium binning, and 30-s acquisition.

### In Vivo Imaging Experiments

Animal experiments were reviewed and approved by the Bioethics Committee of Leiden University, the Netherlands. Eight-wk-old CD-1 nude mice were used for experiments. For initial assessment of fluorescent protein sensitivity, 1 × 10^6^ HEK-293 cells transfected with pTurboLuc, pluc2-iRFP720, and pluc2-iRFP670 were injected subcutaneously. Fluorescence signals were measured using an IVIS Spectrum by the following filter settings (TurboLuc ex/em 570/640 nm, luc2-iRFP670 ex/em 640/680 nm, and luc2-iRFP720 ex/em 710/760 nm). Then, different amounts of hMSCs (1 × 10^6^, 1 × 10^5^, 1 × 10^4^, and 1 × 10^3^ cells) were implanted into the cortex of the mouse to check optical imaging sensitivity using the novel fusion reporter. In brief, mice were anesthetized using isofluorane (Piramal Critical, Bethlehem, PA, USA) and placed in a robot stereotactic device (Neurostar, Tubingen, Germany). Mouse skulls were drilled using this system, and cells were injected at a volume of 2 μL into the cortex at 1 mm depth (Bregma coordinates: AP +0.5; L ± 2.0; DV −1.0). The subsequent day, a dose of 25 μM/kg of D-Luc was injected intraperitoneally (i.p.) 15 min prior to imaging at the IVIS Spectrum imager (Perkin Elmer, Waltham, MA, USA) using an open filter setting and 30-s acquisition time. iRFP720 fluorescent protein emission was measured using the Pearl Imaging system (LICOR Biosciences) at the 700 nm setting. In another set of experiments, 1 × 10^5^ luc2-iRFP720 expressing hMSCs were implanted at 1 mm depth in the cortex, and performances of different luciferin substrates were evaluated, which were 25 μM/kg and 943 μM/kg of D-Luc potassium salt (Promega) and 25 μM/kg of CycLuc1 (Aobious, Gloucester, MA, USA). Substrates were then injected (i.p.) before imaging, and signals were collected when emission peaked. Cells were followed for the subsequent 7 wk using the imaging protocol determined from previous experiments.

### Statistics

Statistical analyses for the experiments (hMSC dilution series for BLI and FLI (Fig. 5), different doses of D-luc and CycLuc1 (Fig. 6), and longitudinal optical monitoring of transplanted hMSCs) were performed using a 1-way analysis of variance with multiple comparisons using Tukey’s post hoc test. Data were analyzed using GraphPad Prism software (GraphPad, La Jolla, CA, USA).

## Results

### Characterization of Fusion Proteins

**Fig. 1.**
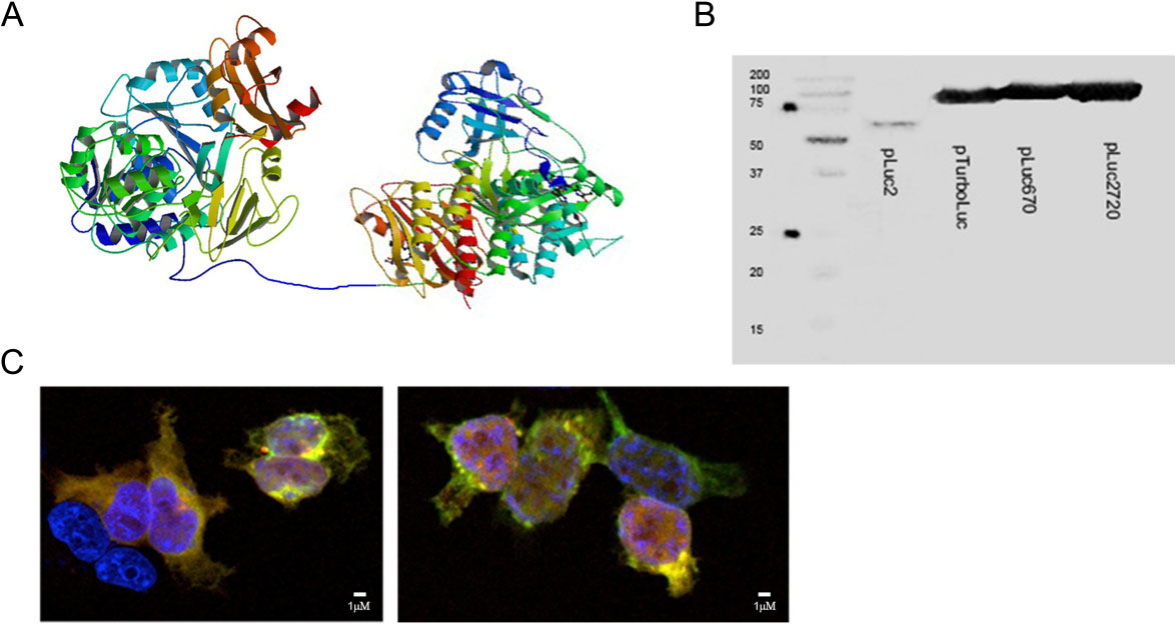
Generation of new fusion luciferase (luc2) reporters with near infrared fluorescence proteins. (A) Schematic drawing of the luc2 fusion with iRFP720 via a 14-aa linker. Photinus pyralis luc2 (PDB ID code 1LCI) and Chromophore-binding domain (CBD) of bacterial phytochrome RpBphP2 (PDB ID code 4E04) were used. (B) Western blot results using an anti-luc2 primary antibody on human embryonic kidney (HEK)-293 lysates used for transient expression of the proteins. The band in the first lane corresponds to luc2 protein (62 KDa), the second to the TurboLuc protein (∼88 KDa). The third and fourth lanes correspond to luc2-iRFP670 (abbreviated pLuc670) and luc2-iRFP720 (abbreviated pLuc2720; ∼97 KDa). (C) Immunofluorescence for the analysis of colocalization of luc2 (green signal) and near infrared proteins (red signal) in HEK-293 cells. Nuclei are stained with 4′,6-diamidino-2-phenylindole (blue signal). luc2 was detected using an anti-luc2 antibody and Fluorescein isothiocyanate (FITC)-labeled secondary antibody. Near infrared proteins were detected using a 633-nM excitation laser. Image on the left corresponds to cells expressing luc2-iRFP670 and image on the right corresponds to cells expressing luc2-iRFP720.

**Fig. 2.**
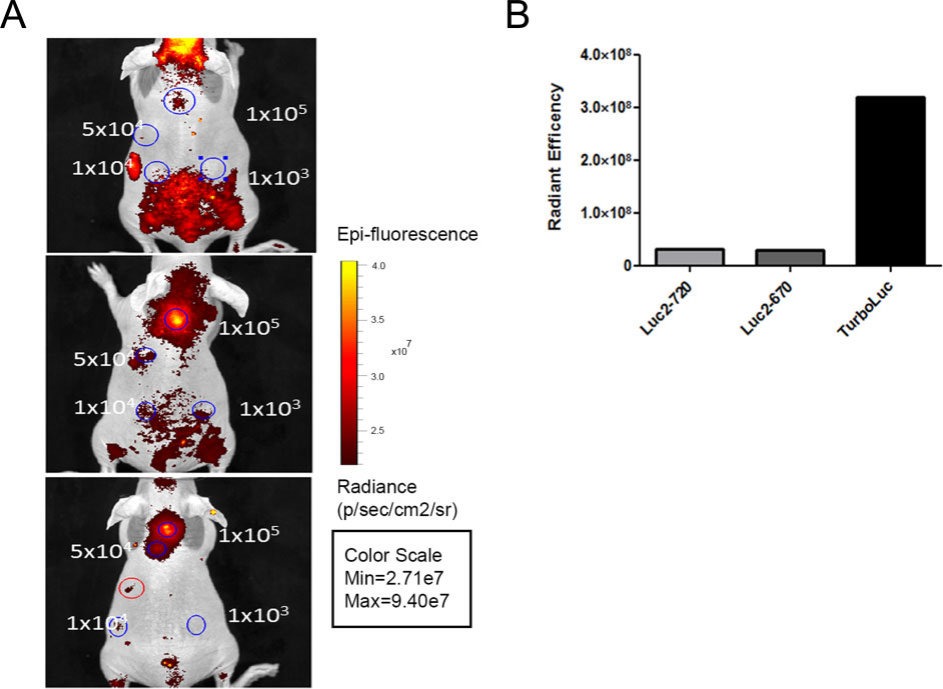
In vitro and in vivo characterization of different fusion proteins. (A) In vivo fluorescence imaging of different amounts of human embryonic kidney-293 cells (blue circles) expressing TurboLuc (upper panel), luc2-iRFP670 (middle panel), and luc2-iRFP720 (lower panel). Red circle indicates autofluorescent food signals from stomach. (B) Quantification of the signal given by 1 × 10^5^ cells. TurboLuc expressing cells show higher total signal. However, background signals are lower when using iRFP720 protein. Data are represented as mean (SD) (*n* = 3).

The novel proteins luc2-iRFP720 and luc2-iRFP670 were fused using the same 14 amino acid linker (Fig. 1A) used to create the TurboLuc protein.^20^ In addition, Western blot analysis was performed to confirm the correct expression of the fused proteins. luc2-iRFP720 and luc2-iRFP670 bands (∼97 KDa) were localized above both luc2 (62KDa) and TurboLuc (88KDa) bands (Fig. 1B). The new fusion proteins were efficiently expressed in HEK-293 cells where colocalization of luc2 and iRFP signals was observed using fluorescence microscopy (Fig. 1C). Decreasing amounts of HEK-293 cells expressing the fused proteins were then implanted subcutaneously in nude mice to check for sensitivity. Although luc2-iRFP720 expressing cells gave low total fluorescent signals in vivo, 1 × 10^4^ cells were detectable while only 1 × 10^5^ cells were detectable using luc2-iRFP670 and TurboLuc (Fig. 2A, B), thanks to an advantageous signal to noise ratio.

### Proliferation of Transduced Human Mesenchymal Stem Cells

**Fig. 3.**
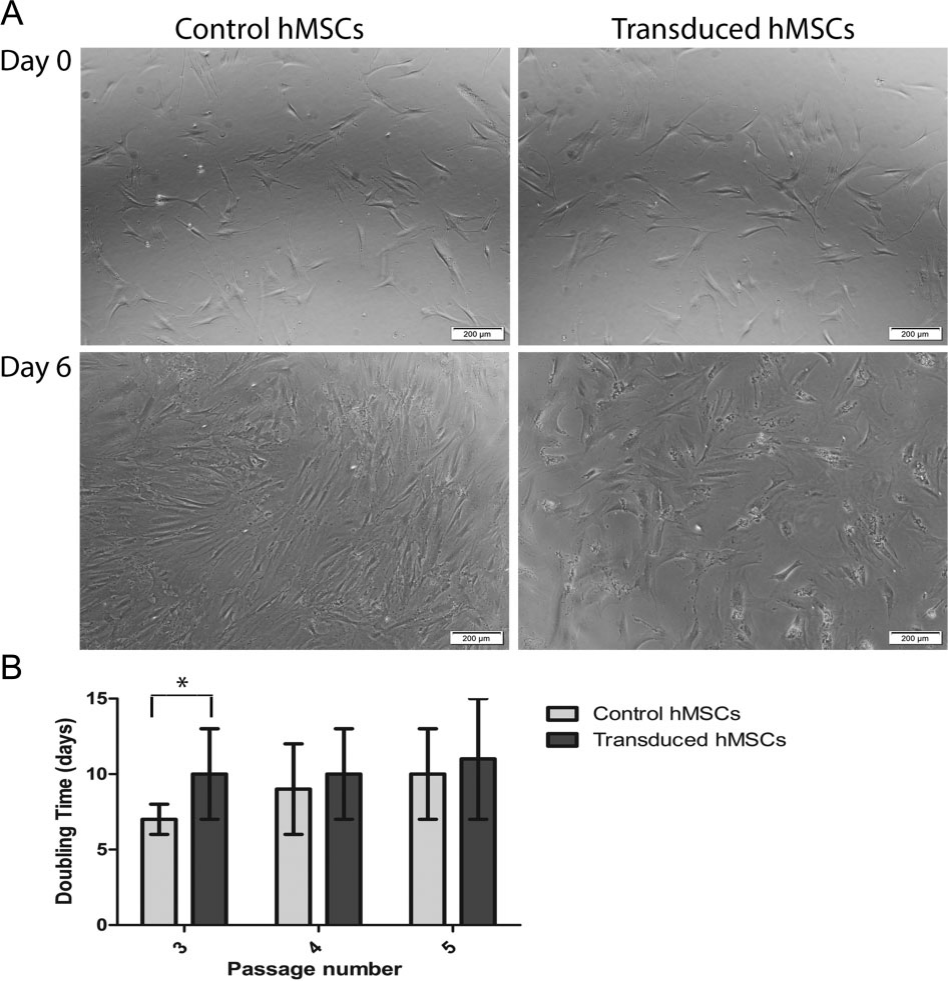
Effect of transduction on proliferation of human mesenchymal stem cells (hMSCs). (A) White-field microscopic images of hMSCs the day before transduction (day 0, upper panel) and 6 d after transduction (lower panel) compared to non transduced cells used as controls. (B) Graph reporting the calculated doubling time of control and transduced hMSCs. Transduced cells at passage 3 show a significantly higher doubling time (*P < 0.05), but no significant differences in the 2 subsequent passages.

Proliferation of hMSCs was evaluated after transduction for 2 consecutive passages (passage 3 to 5). Six days after transduction, cells observed under a microscope clearly showed inhibited proliferation compared to control, non transduced cells (Fig. 3A). Transduced cells had a doubling time of 10 ± 3 d significantly higher than non transduced cells at that passage (7 ± 1 d; *P* < 0.05). After 2 passages, transduced cells showed a doubling time of 11 ± 3 d comparable to non transduced cells that had an average doubling time of 10 ± 3 d (Fig. 3B).

### Differentiation Ability of Transduced Human Mesenchymal Stem Cells

**Fig. 4.**
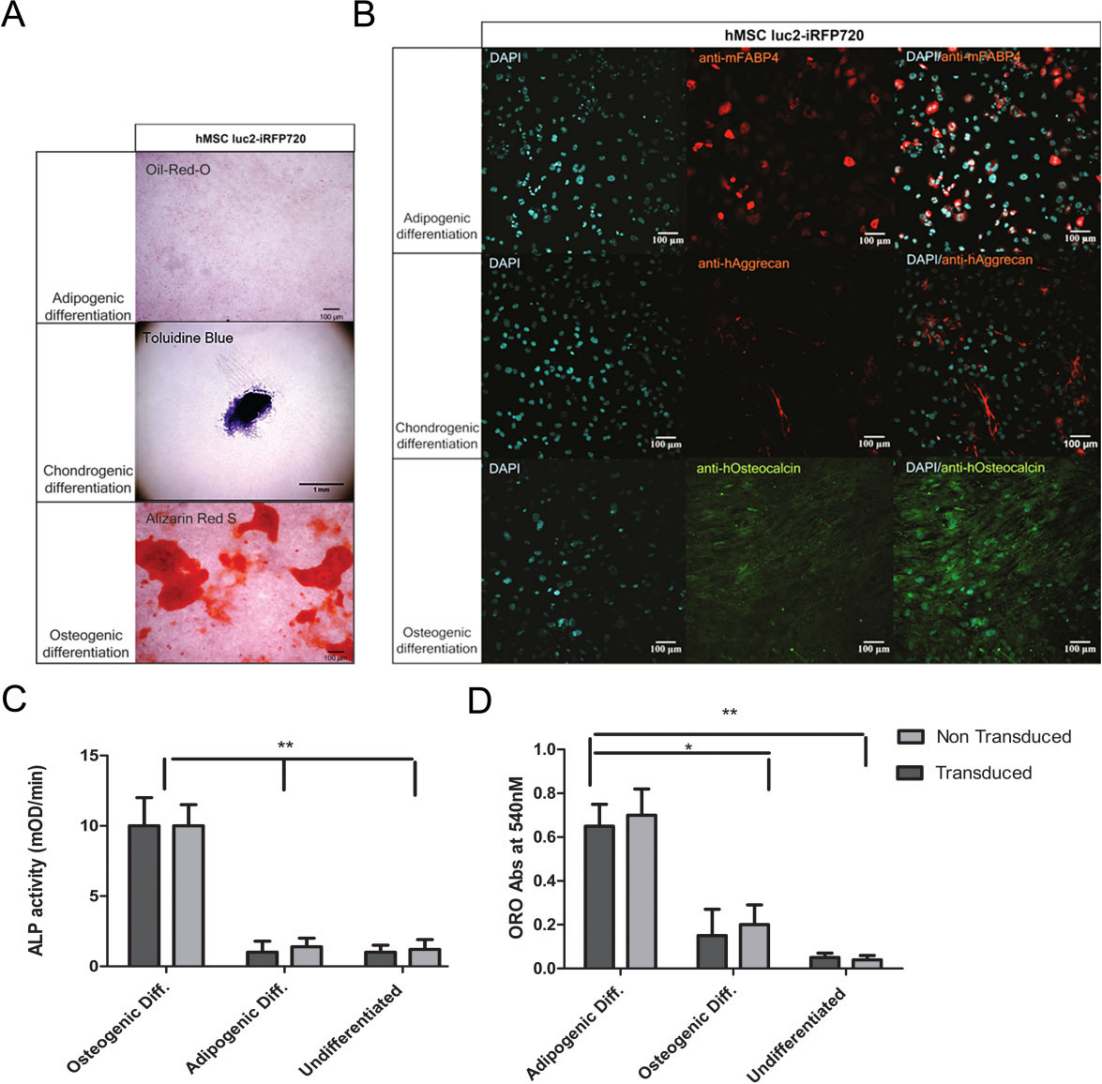
Histological staining and immunofluorescent staining of luc2-iRFP720 expressing human mesenchymal stem cells (hMSCs) in determining differentiation capabilities. (A) Oil Red O staining showed the presence of fat deposition, toluidine blue staining showed the presence of proteoglycans, and alizarin Red S showed the presence of calcific deposition after adipogenic, chondrogenic, and osteogenic differentiation of luc2-iRFP720 expressing hMSCs, respectively. (B; left column) 4′,6-diamidino-2-phenylindole (DAPI) staining showed cell nuclei, (middle column) hMSC luc2-iRFP720 showed immunofluorescent staining of anti-mouse fatty acid binding protein 4 (anti-mFABP4), anti-human aggrecan (anti-hAggrecan), and anti-human osteocalcin (anti-hOsteocalcin) after conditioned adipogenic, chondrogenic, and osteogenic differentiation, respectively. (Right column) Merged images of DAPI and the specific antibody (*n* = 3). (C) alkaline phosphatase (ALP) activity measured in medium of luc2-iRFP720 expressing hMSCs, and non transduced hMSCs after 14 d of adipogenic or osteogenic ALP activity during osteogenic differentiation are significantly higher (*P* < 0.01) than during adipogenic differentiation or undifferentiated control cells. (D) Oil Red O absorbance values at 540 nM suggest that the level of adipocytes in different culturing conditions is not affected by transduction, but it is significantly higher in adipogenic differentiation compared to osteogenic differentiation or undifferentiated cells (*P < 0.05 and **P < 0.01).

Transduced cells were sorted for positive iRFP720 expression using FACS and subsequently expanded and passaged for 3 times. At this time, hMSCs were stimulated to differentiate. luc2-iRFP720 expressing hMSCs were able to differentiate into adipogenic, osteogenic, and chondrogenic cells as observed by the positive Oil Red O staining, Alizarin Red staining, and toluidine staining, respectively (Fig. 4A). The differentiation ability was further verified using specific markers for the different cell lineages. In particular, cells were positive for the adipogenic marker fatty acid–binding protein (FABP4), chondrogenic marker aggrecan, and osteogenic marker osteocalcin following a 14-d differentiation protocol (Fig. 4B). No differences in differentiation capabilities were observed when compared to non transduced hMSCs at the same passage (data not shown).

We further characterized the formation of osteoblasts when performing adipogenic differentiation measuring ALP in medium for transduced and non transduced hMSCs. Our results indicate that osteoblasts were not present during adipogenic differentiation (Fig. 4C).

On the other hand, adipocytes were present during osteogenic differentiation (20% to 25% of the absorbance value at 540 nM) as reflected by the result of relative Oil Red O accumulation using both transduced and non transduced cells (Fig. 4D).

### Sensitivity of In Vivo Fluorescence and Bioluminescence Stem Cell Imaging

**Fig. 5.**
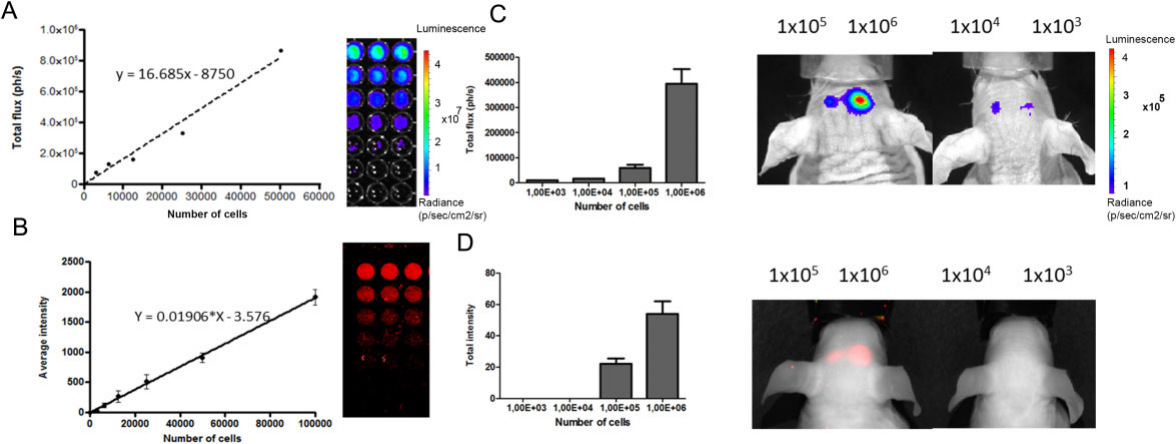
Serial dilutions of luc2-iRFP720 expressing human mesenchymal stem cells (hMSCs) measured for bioluminescence and fluorescence intensity. (A) Bioluminescence imaging (BLI) was measured for the serial dilution of of luc2-iRFP720 expressing hMSCs (0, 3.125, 6.250, 12.500, 25.000, and 50.000 cells; *r* ^2^ = 0.9921). Data are represented as mean (SD) (*n* = 3). (B) fluorescence imaging (FLI) was measured for the serial dilution (0, 3.125, 6.250, 12.500, 25.000, 50.000, and 100.000 cells) at 700 nm (*r* ^2^ = 0.9980) using the Odyssey scanner. Data are represented as mean (SD) (*n* = 6). (C) BLI of decreased amount of cells (1 × 10^3^, 1 × 10^4^, 1 × 10^5^, and 1 × 10^6^). Images were taken 20 min after injection of D-luciferin (25 µM/kg). (D) FLI of decreased amounts of cells (1 × 10^3^, 1 × 10^4^, 1 × 10^5^, and 1 × 10^6^) when injected into the mouse brain using Pearl Imager with 700 nm settings.

To evaluate the sensitivity of stem cell detection in vivo, luc2-iRFP720 expressing hMSCs were implanted in the mouse cortex. First, cell lines were characterized for their emission properties in vitro. As expected, serial dilutions of cells showed a linear correlation with both bioluminescence and fluorescence signals. In vitro experiments using the imaging conditions described in the Materials and Methods section showed that luc2-iRFP720 expressing hMSCs emit an average of 17 photons per second (ph/s) per cell and 0.019 relative light unit per cell of fluorescence (Fig. 5A, B). The in vivo detection limit for BLI was 1 × 10^3^ cells, while for FLI, the in vivo detection limit was 1 × 10^5^ luc2-iRFP720 expressing hMSCs (Fig. 5C, D).

### Longitudinal Monitoring of Transduced Human Mesenchymal Stem Cells in Mouse Brain

**Fig. 6.**
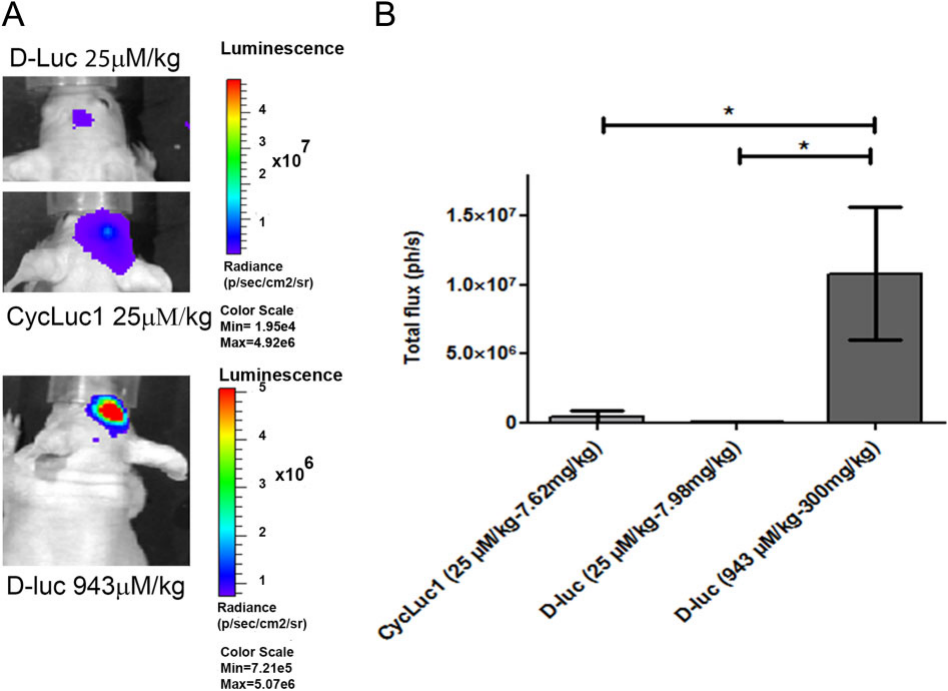
Bioluminescence imaging (BLI) signal in vivo of D-luciferin (D-Luc; 25 µM/kg), CycLuc1 (25 µM/kg), and D-Luc (943 µM/kg) in the mouse brain. (A; top) Injected with D-Luc (25 µM/kg), (middle) injected with CycLuc1 (25 µM/kg), and (bottom) injected with D-Luc (943 µM/kg) in mouse brain. (B) The graph showed that D-Luc (943 µM/kg) gives significantly higher BLI signals (∼1 × 10^7^) than D-Luc and CycLuc1 (25 µM/kg). Data are represented as mean (SD) (*n* = 3; *P ≤ 0.05).

**Fig. 7.**
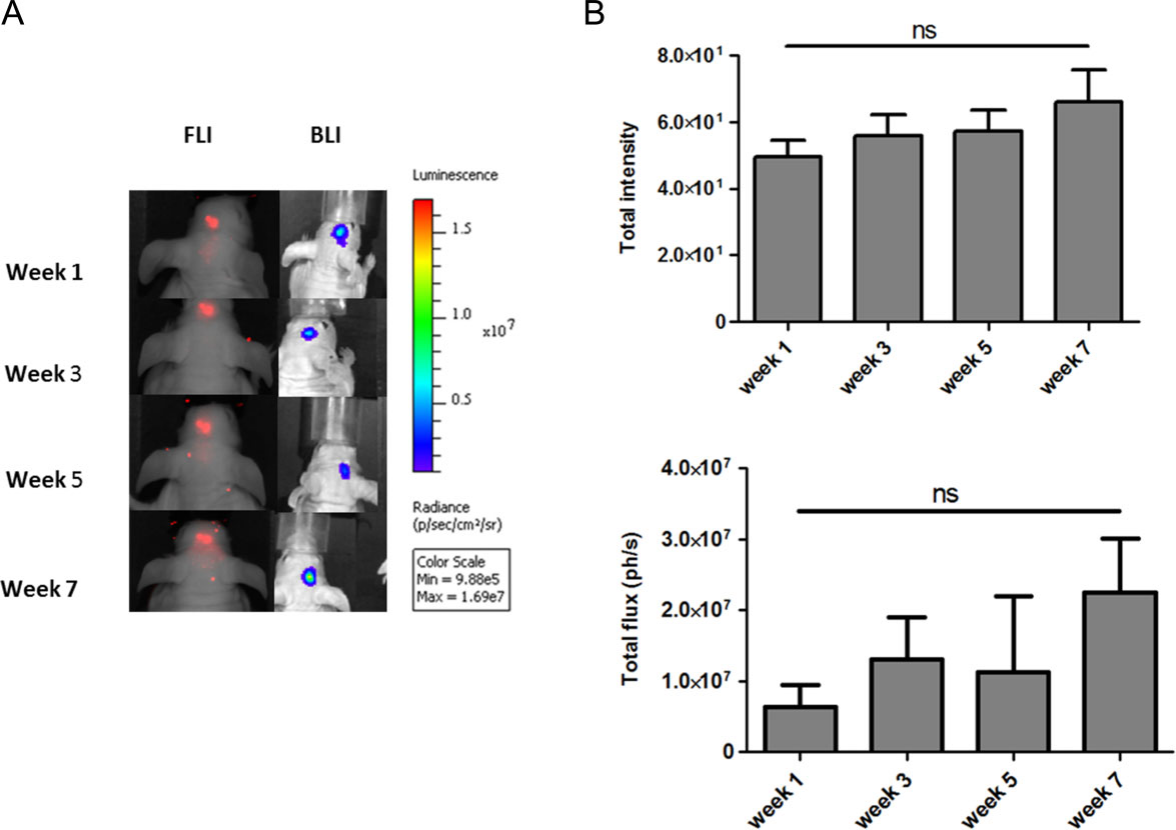
(A) Longitudinal monitoring of luc2-iRFP720 expressing human mesenchymal stem cells when injected in the mouse brain followed for 1, 3, 5, and 7 wk. (B) No significant differences in bioluminescence imaging of fluorescence imaging signal were observed between the weeks. Data are represented as mean (SD) (*n* = 4; **P < 0.01 and *P < 0.05).

For longitudinal monitoring of luc2-iRFP720 expressing hMSCs, 1 × 10^5^ cells were transplanted into the cortex of nude mice with initial high signals in order to maintain sensitivity of detection over time. In our experiments, different BLI protocols were evaluated, wherein different luciferin substrates and doses were applied. D-Luc (943 µM/kg) showed a significant higher signal with an average of 1 × 10^7^ ph/s when compared to CycLuc1 (25 µM/kg) and D-Luc (25 µM/kg) in mouse brain (*P* ≤ 0.05; Fig. 6A, B). For that reason, D-Luc (943 µM/kg) was utilized for the longitudinal monitoring experiment, wherein BLI signals of luc2-iRFP720 expressing hMSCs were measured in the mouse brain. luc2-iRFP720 expressing hMSCs could still be detected after a period of 7 wk in the mouse brain (Fig. 7A) with an average of ∼1.3 × 10^7^ ph/s and showed no statistically significant differences in BLI or FLI signals between the weeks (Fig. 7B, C).

## Discussion

The main goal of this research was to develop a genetically encoded reporter for multimodal optical imaging that would allow for longitudinal monitoring of transplanted stem cells with high sensitivity. We fused the codon-optimized firefly luc2 and NIRFPs that were previously reported to be the most sensitive reporters for deep tissue imaging.^15,18^ To fuse the reporters, we opted for the 14-aa linker previously employed in the generation of TurboLuc protein.^20^ The novel fusion reporters luc2-iRFP670 and luc2-iRFP720 were successfully expressed in HEK-293 cells. Moreover, subcutaneous transplantation of transiently transfected HEK-293 cells in nude mice revealed that iRFP720 has a substantially lower background compared to iRFP670 and pTurboLuc, making it superior to iRFP670 for imaging purposes as shown already by Luker et al.^18^ Therefore, bone marrow–derived hMSCs were transduced by a lentivirus expressing luc2-iRFP720. However, before proceeding with in vivo experiments, we evaluated whether transduction had an effect on cell proliferation or whether the expression of the novel fusion reporter would affect the differentiation capabilities of hMSCs. Our results demonstrated that the transduced hMSCs had a significantly lower doubling time compared to non transduced control cells at passage 3, but after consecutive passages, the doubling time was not significantly different (passage 5). The effect of lentiviral transduction using polybrene has been previously reported for MSCs at first passage.^22,23^ In addition, transduced hMSCs were able to differentiate into chondrocytes, adipocytes, and osteocytes in accordance with previous reports, and no ill effects were observed.^24,25^ This is especially important if hMSCs are under investigation for regenerative purposes. We further characterized the level of the expression of the bioluminescent and fluorescent reporter and evaluated the sensitivity of detection in vivo. The level of expression is dependent on the choice of the promoter. The PGK promoter is commonly used to express proteins in hMSCs.^26^ We are aware that other constitutive promoters like elongation factor 1 would allow an even better expression, but no direct comparisons have been reported in literature yet.^27,28^ Analysis and comparison of the BLI and FLI performances in vivo were possible, since fusion of the reporters ensured equimolar expression in cells. Our results showed that the limit of detection using BLI is approximately 1 × 10^3^ hMSCs in the brain cortex using a dose of 25 μM/kg of D-Luc. Previous report showed that 3 × 10^3^ stem cells were visible using a dose of 943 µM/kg of D-Luc.^29^ Since transduced stem cells emitted an average of 17 ph/s per cell in vitro using our conditions, it is likely that cells with higher photon fluxes would be able to lower the limit of detection in vivo. On the contrary, the limit of detection using FLI is around 1 × 10^5^ cells. Although BLI has proven to be more sensitive, our results support the use of iRFP720 fluorescent protein for applications where higher amounts of cells are transplanted. To date, the iRFP720 reporter has been used only for monitoring tumor progression using FLI.^30^ In addition, iRFP720 has shown potential for photoacoustic imaging of tumor progression.^31^ However, in both applications, millions of cells need to be transplanted. To achieve further sensitivity using BLI, we explored the use of different D-Luc substrates and doses after i.p. injection. In fact, it is known that D-Luc can pass the blood–brain barrier (BBB), but the concentration of D-Luc in the brain following i.p. injection of a dose of 422 µM/kg (generally considered as a standard) is far from being saturating and it can be further increased and well tolerated.^32^ Aswendt et al., for example, reported superior performances for imaging transplanted stem cells with a D-Luc dose of 943 µM/kg.^29^ On the other hand, a dose of 25 μM/kg of CycLuc1 was reported to enhance imaging sensitivity of lentivirally transduced endogenous cells in the mouse brain when compared to the standard dose of D-Luc of 422 µM/kg.^16^ Considering these recent reports, we aimed to compare a dose of 25 μM/kg of CycLuc1 to a dose of 25 μM/kg and 943 µM/kg of D-Luc. Using a dose of 943 µM/kg of CycLuc1 is not possible due to the limited solubility of the compound (maximum solubility 100 mg/mL in dimethyl sulfoxide [DMSO] that allows injecting a maximum of 160 μM/kg i.p. in 5% DMSO).^16^ Importantly, signals were compared when emissions peaked after substrate injection. In particular, experiments were carried out 1 wk after cell transplantation when the BBB was restored to take into account the different influx of D-Luc and CycLuc1 when the BBB was breached. Our results demonstrated that using a higher dose of D-Luc was able to generate brighter signals in brain cortex than 25 μM/kg of CycLuc.^16^ Following this protocol, initially transplanted 1 × 10^5^ hMSCs could be detected in the cortex of the mouse brain for 7 wk without any substantial drop in BLI signals, analogous to the findings of Tennstaedt et al., wherein 3 × 10^5^ human neuronal stem cells could be detected in the cortices of mice for several weeks.^33^ Remarkably, the generated photon flux (ph/s) was about 10^7^ ph/s, a flux higher than previously reported for imaging transplanted stem cells at this depth.^11,15,30,33–35^

In addition, cells could be detected using FLI of the iRFP720 protein. To the best of our knowledge, there are no other reports where less than 10^5^ hMSCs could be detected with adequate sensitivity and resolution in the brain cortex for several weeks using optical imaging. In conclusion, the 3 pending questions have found an answer. The novel fusion reporter showed that BLI allows for more sensitive imaging in the brain than iRFP720. The latter remains a good option for imaging an amount of cells higher than 1 × 10^5^. A concentration of D-Luc of 943 µM/kg allows for the efficient tracking of transplanted stem cells in the mouse brain using optical imaging for at least 7 wk.
